# Bioconcentration and Acute Intoxication of Brazilian Freshwater Fishes by the Methyl Parathion Organophosphate Pesticide

**DOI:** 10.1155/2015/197196

**Published:** 2015-08-03

**Authors:** João Bosco de Salles, Renato Matos Lopes, Cristiane M. C. de Salles, Vicente P. F. Cassano, Manildo Marcião de Oliveira, Vera L. F. Cunha Bastos, Jayme Cunha Bastos

**Affiliations:** ^1^Universidade Estadual da Zona Oeste, Rio de Janeiro, RJ, Brazil; ^2^Laboratório de Comunicação Celular, Fundação Oswaldo Cruz, Instituto Oswaldo Cruz, Avenue Brasil 4365, Manguinhos, 21045-900 Rio de Janeiro, RJ, Brazil; ^3^Setor de Bioquímica, Departamento de Química, Universidade Federal Rural do Rio de Janeiro, Seropédica, RJ, Brazil; ^4^Department of Biology, Loyola Marymount University, Los Angeles, CA, USA; ^5^Laboratório de Ecotoxicologia e Microbiologia Ambiental (LEMAM), Instituto Federal de Educação, Ciência e Tecnologia Fluminense, Cabo Frio, RJ, Brazil; ^6^Departamento de Bioquímica, Instituto de Biologia Roberto Alcântara Gomes, Universidade do Estado do Rio de Janeiro, Rio de Janeiro, RJ, Brazil

## Abstract

Three species of freshwater Brazilian fishes (pacu, *Piaractus mesopotamicus*; piavussu, *Leporinus macrocephalus*, and curimbatá, *Prochilodus lineatus*) were exposed to an acute dose of 5 ppm methyl parathion organophosphate pesticide. Three to five individuals per species were exposed, one at a time, to 40 liters tap water spiked with Folidol 600. Pesticide concentrations and cholinesterase (ChE) activities were evaluated in serum, liver, brain, heart, and muscle. The bioconcentration of methyl parathion was similar for all studied fishes. Brain tissue showed the highest pesticide concentration, reaching 80 ppm after exposure for 30 min to methyl parathion. Three to 5 hours of 5 ppm methyl parathion exposure provoked the death of all *P. lineatus* at 92% brain AChE inhibition, whereas fish from the other two species survived for up to 78 hours with less than 80% brain AChE inhibition. Our results indicate that acute toxic effects of methyl parathion to fish are correlated with brain AChE sensitivity to methyl paraoxon.

## 1. Introduction

Pesticides are a group of toxic compounds with a deep effect on aquatic life and water quality. Organophosphates (OP) are a group of pesticides widely used in Latin America. For instance, in Brazil, methyl parathion (O,O-dimethyl O-p-nitrophenylphosphorothioate) has extensively been applied in agriculture, food storage shelters, pest control programs, and aquaculture ponds to control aquatic insect larvae, which are predators of fingerling [1, 2]. The development of the Brazilian freshwater fish industry was accompanied by the extensive use of methyl parathion to control ectoparasite infestations in fish. This practice resulted in the discharge of large amounts of methyl parathion-treated waters into the nearby area. Since OP has no selectivity for any specific target organism, discharged waters might cause the intoxication of natural populations of aquatic organisms [3, 4].

An important mechanism of acute toxicity by methyl parathion is the inhibition of acetylcholinesterase (AChE) activity. Methyl parathion is a weak acetylcholinesterase inhibitor, but it can be activated into a more potent metabolite, its oxon derivative, methyl paraoxon (O,O-dimethyl O-p-nitrophenyl phosphate), by a desulfuration reaction catalyzed by cytochrome P-450 [5]. Methyl parathion is metabolized by both plants and animals and it is not expected to persist or bioconcentrate. However, studies have reported that* Girardinichthys multiradiatus *captured in the Ignacio Ramirez Dam (Mexico) accumulated methyl parathion more than 13,000 times in relation to water levels of this compound [6, 7]. Methyl parathion can be detoxified by dealkylation by glutathione S-transferases (GST) [8], whereas methyl paraoxon can be removed from blood by scavenger enzymes, such as carboxylesterase and cholinesterases, or degraded by paraoxonase, yielding 4-nitrophenol and dimethylphosphoric acid [9].

Intoxication mechanisms of aquatic organisms by OP are not completely understood. Dembéle and coworkers have attributed fish death to asphyxia resulting from gill irritation, not dependent on brain AChE inhibition [10]. Patil and David [11] reported that sublethal OP levels could induce oxidative stress and inferred that oxidative damages might be related to the death of exposed animals. Nevertheless, OP toxicity can be attributed to AChE inhibition. In neuromuscular junctions and the central nervous system, AChE inhibition induces excessive cholinergic stimulation, producing in coordination, fatigue, involuntary muscle contractions, and eventually, paralysis of the body extremities and the respiratory muscles [12]. Accordingly, kinetic characterization of AChE and butyrylcholinesterase (BChE) (another cholinesterase form) allows the determination of sensitivity differences between both enzymes to organophosphate pesticides, which is essential for environmental monitoring programs [4, 13]. Therefore, the aim of this work was to study the effects of organophosphate methyl parathion on ChE activities from different tissues and organs from three freshwater fish species that dwell in Brazilian waters.

## 2. Material and Methods

### 2.1. Chemicals

Acetylthiocholine iodide (ASCho), butyrylthiocholine iodide (BSCho), propionylthiocholine iodide (PrSCho),* p*-nitrophenol (PNP), 5,5′-dithiobis (2-nitrobenzoic acid) (DTNB), and methyl paraoxon (O,O-dimethyl O-p-nitrophenyl phosphate) were obtained from Sigma (St. Louis, MO, USA). Triton X-100 was acquired from Riedel of Haën AG (Hannover). Isooctane HPLC grade was acquired from Merck. Folidol 600 (methyl parathion 600 g L^−1^, Bayer-Brazil) was purchased at the local market. All other reagents were of analytical grade.

### 2.2. Animals and Experimental Procedures

Common and scientific names, geographical origin, and size of fishes examined in this work are shown in Table 1. Specimens of* Piaractus mesopotamicus* (Holmberg, 1887), commonly named pacu, and* Leporinus macrocephalus* Garavello & Britski, 1988 (piavussu), were supplied by the Morro Grande Fish Farm, RJ, Brazil. Specimens of curimbatá,* Prochilodus lineatus* (Valenciennes, 1836), were supplied by the Sol Nascente Fish Farm, RJ, Brazil. Experimental procedures were carried out according to the ethical principles of animal experimentation elaborated by the Brazilian College for Animal Experimentation (COBEA), which is in agreement with the uniform requirements for manuscript submissions to biomedical journals.

Prior to the assays, the fish were separated by species and kept into 500 L tanks with dechlorinated water at 25 ± 2°C for 15 days. Water was constantly aerated through a biological filtering system pushed by common pumps to produce 5 mg O_2_ L^−1^ (measured with an oximeter). Fish were always on a 14 : 10-h light/dark cycle and fed with a commercial pellet once a day at 9 o'clock AM. Water pH was 6.4 ± 0.2.

In order to be exposed fish were removed from the larger tanks and individually kept in 40 L aquaria filled with water obtained from the 500 L tanks. Methyl parathion (as Folidol) was added once to a final 5 ppm concentration. All fish were not fed 24 h prior to and during the experiments in order to limit organic matter in the water. Moreover, the water during fish exposure was not filtered to avoid removal of the pesticide. Control fish were submitted to the same conditions, without Folidol.

The concentration of 5 ppm methyl parathion was chosen to elicit a quick response and to correlate the inhibition of cholinesterase with the bioconcentration of pesticide in several tissues and organs of the fish.

The intervals of 30 min, 24 hours, and 78 hours were chosen to carry out laboratory analyses. Curimbatás showed more sensitivity to methyl parathion. This was noticed when they stopped moving their opercula. Once this happened, they were immediately removed and laboratory analyses were immediately carried out.

### 2.3. Tissue Samples

Blood was collected by puncture of the dorsal aorta. Then, the fish were euthanized by quickly sectioning their spinal cord. Liver, brain, and heart were removed using scissors and tongs. Also, a portion of epaxial muscle (1 g wet tissue) was excised. All tissues were separately washed quickly with 50 mL of ice-cold saline (0.9% NaCl) and placed into cryogenic vials that were dropped into liquid nitrogen for storage. They were thawed separately by suspension in four volumes of ice-cold 0.1 mol L^−1^ potassium phosphate buffer, pH 7.0. They were minced with scissors and homogenized with 20 strokes in a Potter-Elvehjem apparatus while being maintained in an ice bath at 5-6°C. ChE assay in brain and liver tissue were carried out using this crude homogenate as sample. Samples for assaying heart and muscle ChE were produced by mixing their homogenates with three volumes of 10 mmol L^−1^ Tris-HCl buffer, pH 7.0, containing 3% Triton X-100 and 1 mol L^−1^ sodium chloride. After centrifuging these mixtures at 3,000 ×g for 10 min at 5°C enzyme assays were carried out in the resulting heart and muscle Triton X-100 soluble supernatant fractions.

### 2.4. Methyl Parathion Determinations

Serum and homogenate samples (200 *μ*L) were extracted by using a mixture of 6 mL of isooctane, 200 *μ*L methanol, and 200 *μ*L saturated sodium chloride solution. The extracts were centrifuged at 3,000 ×g for 10 min at 5°C. Three milliliters from each supernatant fraction was collected and evaporated under a gentle nitrogen stream. The residue was reconstituted in 200 *μ*L of acetonitrile. From this reconstituted sample, 50 *μ*L were injected onto a 200 mm × 4.6 mm ODS Hypersil RP-18, 5 *μ*m particle size HPLC column, using (50 : 50 v/v) acetonitrile/ultrapure water as mobile phase with a flow-rate of 1 mL min^−1^, and examined under UV light with the detector set at 270 nm. Under these conditions, methyl parathion recovery was estimated at approximately 92%, following quantification based on a standard prepared with a 98% methyl parathion pure sample previously obtained by thin-layer chromatography.

### 2.5. Cholinesterase Assays

Serum and tissue homogenate samples were placed in a medium containing 1.8 mmol L^−1^ from one of the three substrates (acetylthiocholine, propionylthiocholine, or butyrylthiocholine), with 0.32 mmol L^−1^ DTNB. Enzyme activity was continuously recorded up to 90 s, at 412 nm, using a Shimadzu spectrophotometer, model UV-160 A, according to the Ellman method [14]. The reaction was carried out in microcuvettes. All reagents were dissolved to 200 *μ*L final volume with a 0.1 mmol L^−1^ sodium phosphate buffer, pH 7.5, at 25°C, and the thionitrobenzoate ion concentration was estimated using an extinction coefficient of 14,150 M^−1 ^cm^−1^. One unit (1 U) of enzyme activity was defined as the amount that hydrolyzes 1.0 *μ*mol of substrate per minute.

## 3. Results

### 3.1. Tissue Distribution of ChE Activity

**Figure 1:**
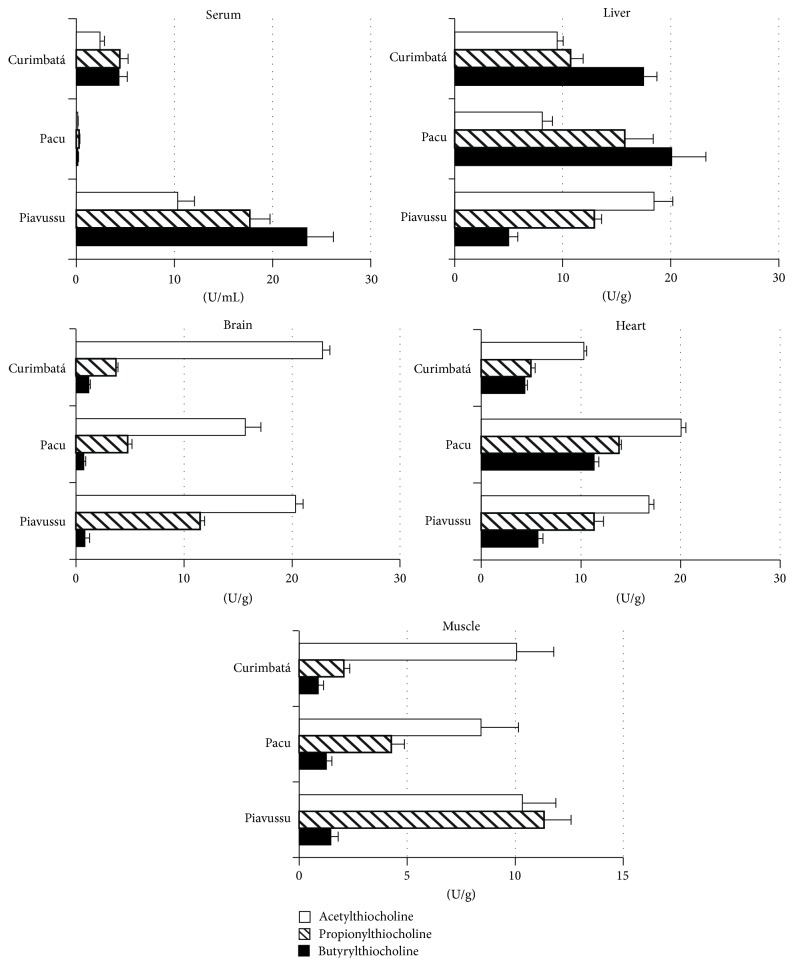
Tissue distribution of cholinesterase activity in pacu, piavussu and curimbatá (control fish). The box shows the substrates used in enzyme assays. Results are expressed as *μ*mol of products formed per minute per gram of wet tissue (U g^−1^) or per mL of serum (U mL^−1^). Liver and brain ChE activities were assayed in homogenates, while heart and muscle ChE activities were assayed in the enzymatic fraction solubilized with Triton and NaCl. Results are average values with the corresponding SEM of assays carried out in five individuals of each species.

The studied fish species (control groups) exhibited remarkable differences in tissue-specific cholinesterase activity (Figure 1). The ChE activity measured in the serum of curimbatá and pacu specimens was lower than the activity measured in brain, liver, heart, and muscle. By contrast, in serum of piavussu the ChE activity was similar to that measured in liver, brain, heart, and muscle.

### 3.2. Methyl Parathion Bioconcentration

**Figure 2:**
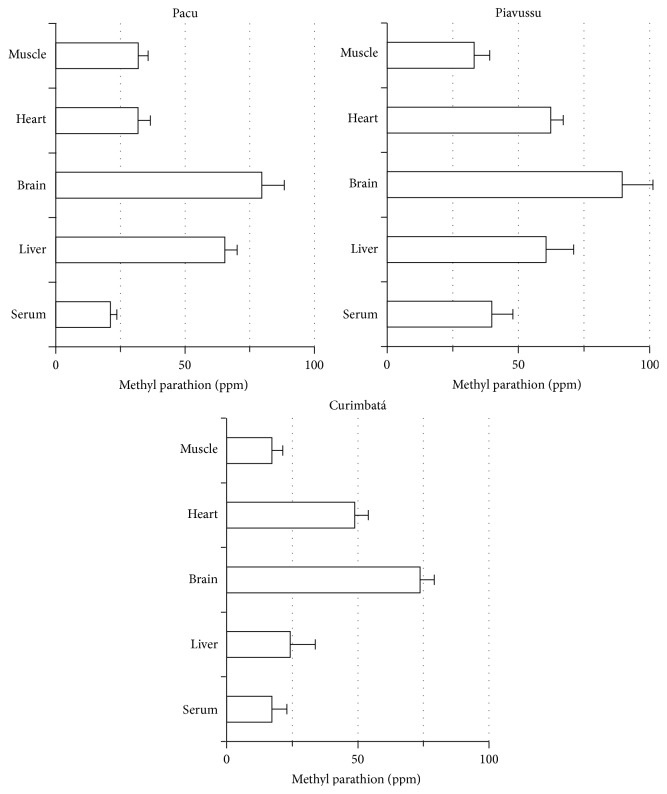
Concentration of methyl parathion in muscle, heart, brain, and liver and serum from pacu, piavussu, and curimbatá. Each one of four animals per species was placed in separated tanks containing 40 L of water with Folidol to produce 5 ppm methyl parathion. After 30 minutes exposure, blood samples were collected and the fishes were euthanized. Tissues were dissected and homogenized. Methyl parathion was extracted from serum and tissue homogenates and quantified by HPLC.

**Figure 3:**
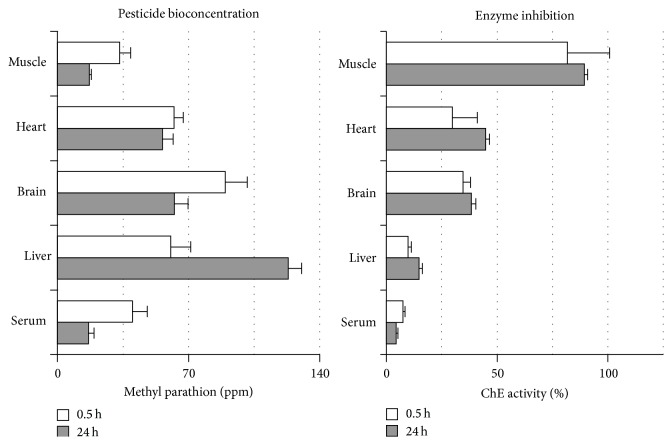
Piavussu methyl parathion concentrations and cholinesterase inhibition after exposure. Each one of eight piavussus was individually placed in separated tanks containing 40 L of water with Folidol to produce 5 ppm methyl parathion. Incubation happened for the showed times. After exposure, blood was collected, fishes were submitted to euthanasia and tissues were dissected and homogenized. Cholinesterase activity was assayed in serum and in homogenates tissues with acetylthiocholine and expressed as percentage of control activity. Methyl parathion was extracted from serum and tissue homogenates and quantified by HPLC.

Muscle, heart, brain, liver, and serum from pacu, piavussu, and curimbatá exhibited similar methyl parathion bioconcentration patterns (Figure 2). Brain tissue showed the highest capacity for methyl parathion bioconcentration, reaching 80 ppm (16-fold increase) after 30 minutes exposure to methyl parathion in water (Figure 2). The maximum methyl parathion concentration in muscle, heart, brain, and serum was achieved after 30 minutes, with one exception: methyl parathion concentrations in piavussu showed an increase after 24 hours exposure, reaching a maximum of 120 ppm in liver, corresponding to a 24-fold increase (Figure 3). Methyl parathion was not found in samples from control animals.

### 3.3. Cholinesterase Inhibition

**Figure 4:**
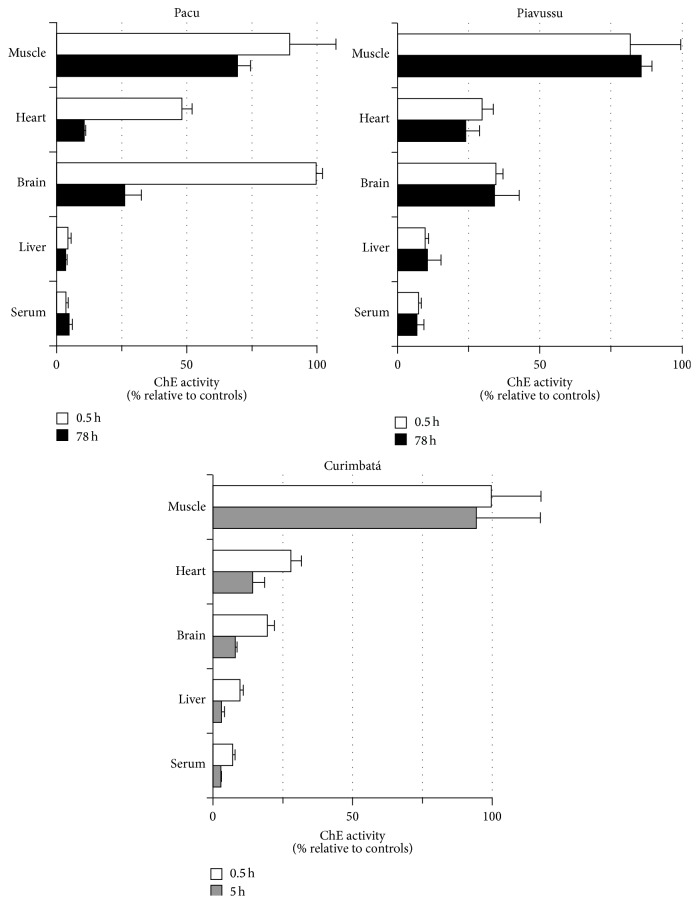
Cholinesterase activity in tissues of pacu, piavussu, and curimbatá exposed to 5 ppm methyl parathion. Each one of eight animals by species was individually treated with Folidol 600 for the indicated time in a 40 L aquarium. After 30 min of exposure blood was collected from four fishes by species and these fishes were submitted to euthanasia. Then, of the remaining fish, four pacus and four piavussus were euthanized after 78 h exposure, while four curimbatás were collected immediately after they showed no movement of opercula (from 3 to 5 h of exposure). Tissues were dissected and homogenized. Cholinesterase was assayed in serum and homogenates with acetylthiocholine and expressed as percentage of the activity found in controls.

Heart, liver, and serum cholinesterase activities for pacu, piavussu, and curimbatá were greatly inhibited after 30 minutes exposure to Folidol. By contrast, skeletal muscle ChE activity for the three species and brain AChE activity for pacu showed no inhibition within this time period (Figure 4). Exposure of fishes to water with Folidol for 78 h resulted in 70% brain AChE activity inhibition in pacu and 60% brain AChE activity inhibition in piavussu.

All curimbatás stopped moving their opercula between 3 and 5 hours of exposure to 5 ppm Folidol, showing more than 90% inhibition of brain AChE activity and no inhibition of skeletal muscle ChE activity (Figure 4). Increasing the time of exposure from 30 min to 78 hours did not considerably modify muscle, heart, brain, liver, or serum ChE activities in piavussu (Figure 4).

## 4. Discussion

Brain tissue showed the highest pesticide bioconcentration, reaching 80 ppm after 30 minutes of exposure to methyl parathion (Figure 2). The three fish species selected for this study present different brain cholinesterase sensitivities to intoxication by methyl paraoxon [15]. In these fishes, the sensitivity of brain ChE to intoxication by methyl paraoxon is inversely related to the activity of the brain enzyme for ASCho [15], in contrast to what was described for trout and rat by Kemp and Wallace [16].

The results demonstrate that the fishes exposed to Folidol rapidly absorbed methyl parathion, since concentrations of methyl parathion in the tissues and serum peaked within 30 min of exposure to Folidol. This concentration continued to increase only in liver, in which the amount of methyl parathion doubled after 24 hours (Figure 3). Such findings can be ascribed to the promptness with which methyl parathion undergoes biotransformation into methyl paraoxon inside liver cells, as compared to other organs. Since P-450 can be inhibited by the sulphur removed from parathion [17, 18] it is plausible that metabolism of methyl parathion molecules occurred in liver up to 30 min and then a consequent inhibition of liver P-450 allowed that more methyl parathion could accumulate and be extracted. When rainbow trout was exposed to the 75 ng mL^−1^ ethyl parathion Abbas and coworkers [19] observed a peak concentration of this pesticide in plasma up to 4.5 hours. In the experiments conducted in the present study, pesticide bioconcentration occurred over a shorter period, probably due to the significantly higher pesticide concentration used. Our results indicate that methyl parathion can bioconcentrate in brain of the tested fish up to approximately 80 ppm (4 times its solubility in water) (Figure 2). This bioconcentration capacity is probably due to high lipid solubility of this OP compound. The half-life of parathion in fish has been reported as 5 times longer than in rats [19]. This suggests that this pesticide could persist for a relatively long time in fish, raising concerns regarding human consumption of fish previously exposed to OP.

The results reported herein demonstrated that ChE inhibition in fish exposed to Folidol does not seem to depend on the methyl parathion bioconcentration in their tissues. No ChE inhibition in pacu brain was observed up to 30 min of exposure, despite the fact that this organ presented 80 ppm of methyl parathion at that time. Therefore, this data is important for further investigations. Tissues in which ChE inhibition occurred more quickly, such as pacu liver (Figure 4) showing higher BChE activity in comparison to AChE activity (Figure 1). BChE has been shown to be more sensitive to methyl paraoxon and is probably more quickly inhibited [20]. In addition, activation of OP compounds occurs mainly in the liver because of the high concentrations of P-450 in that tissue. Thus, liver ChE undergoes inhibition by methyl paraoxon locally generated in tissue before this metabolite leaks out to general blood circulation and reaches other target tissues.

In their work, de Aguiar and coworkers [2] described an 87% brain AChE inhibition in matrinxã (*Brycon cephalus*) exposed to water with 2 ppm Folidol 600 for 96 hours. The present study indicates that piavussu and pacu might be more tolerant to Folidol than matrinxã, since piavussu presented 66% and pacu 74% brain AChE inhibition when exposed to water with 5 ppm methyl parathion for 78 hours. On the other hand, curimbatá showed a 92% brain AChE inhibition with only 5 hours of exposure to 5 ppm methyl parathion, probably due to the higher sensitivity of this species AChE to inhibition by methyl paraoxon [15].

Mammals poisoned by OP usually die by asphyxia. However, fishes have been reported to be more resistant to poisoning by high levels of organophosphates compounds than rats [21]. The actual causes and mechanisms of fish poisoning by OP are not fully understood. Our findings reinforce that interspecific differences in AChE inhibition by their oxon derivatives should be considered. Physiological and behavioral disturbances start at 50% AChE inhibition and death usually follows when inhibition exceeds 80% in mammals and birds [22]. We found here that curimbatás also perished when brain AChE inhibition reached above 80%.

This study suggests that a major contributing factor to acute fish toxicity by Folidol is brain AChE sensitivity to methyl paraoxon, the oxon derivative of methyl parathion. The same explanation, that is, the sensitivity of brain AChE to oxon derivatives, has been suggested for chlorpyrifos, parathion, and methyl parathion toxicity in mosquito fish (*Gambusia affinis*) [23]. Although it was clearly established that curimbatás possess the most sensitive brain AChE among the studied species, the death of curimbatás exposed to 5 ppm methyl parathion cannot be solely credited to brain AChE inhibition. Other possible causes of this fish death, such as Na^+^ K^+^ ATPase inhibition [24] and tissue hypoxia, which compromises heart function, need to be examined.

## 5. Conclusion

*Prochilodus lineatus* (curimbatá),* Piaractus mesopotamicus* (pacu), and* Leporinus macrocephalus* (piavussu) studied here showed similar capacities to bioconcentrate methyl parathion in their tissues after exposure to 5 ppm in water. However, only curimbatá, with the highest brain AChE sensitivity to methyl paraoxon, died after 5 hours of exposure to Folidol. Pacu and piavussu are more resistant to methyl paraoxon and were alive up to 78 hours of exposure to 5 ppm of methyl parathion. Brain AChE sensitivity to methyl paraoxon might be a decisive factor for determining the sensitivity of these species to poisoning by high concentrations of organophosphate compounds. The present study indicates that fishes whose brain acetylcholinesterase activity is more sensitive to oxon derivatives will suffer more severe impacts from environmental contamination by organophosphate pesticides.

Measures reducing the use of organophosphate pesticides in fish culture should be adopted in order to minimize the discharge and consequent impact of these chemicals to natural communities inhabiting rivers and lakes, of which the sensitivity to intoxication by pesticides is largely unknown.

## Figures and Tables

**Table 1:** Common and scientific name, suppliers, and average size of fishes examined.

Common name	Scientific name	Supplier^a^	Size and SD^b^ (cm)
Curimbatá	*Prochilodus lineatus* (Valenciennes, 1836)	Sol Nascente Fish Farm	18 ± 5

Pacu	*Piaractus mesopotamicus* (Holmberg, 1887)	Morro Grande Fish Farm	18 ± 4

Piavussu	*Leporinus macrocephalus* Garavello and Britski, 1988	Morro Grande Fish Farm	20 ± 5

^a^All suppliers are located in the state of Rio de Janeiro, Brazil.

^b^SD: standard deviation.
